# Increased risk of testosterone deficiency is associated with the systemic immune-inflammation index: a population-based cohort study

**DOI:** 10.3389/fendo.2022.974773

**Published:** 2022-08-16

**Authors:** Yongchao Li, Minghui Liu, Yu Cui, Zewu Zhu, Jinbo Chen, Feng Zeng, Meng Gao, Yang Li, Fang Huang, Hequn Chen

**Affiliations:** ^1^ Department of Urology, Xiangya Hospital, Central South University, Changsha, China; ^2^ National Clinical Research Center for Geriatric Disorders, Xiangya Hospital, Central South University, Changsha, China

**Keywords:** testosterone, the systemic immune-inflammation index, testosterone deficiency, NHANES, inflammation

## Abstract

**Purpose:**

This study aimed to explore the relationship between serum testosterone levels and systemic immune-inflammation index (SII).

**Methods:**

Complete SII and serum testosterone data of men over 20 years of age were retrieved from the 2011–2016 National Health and Nutrition Examination Survey to conduct a prevalence survey. To calculate SII, the platelet count was multiplied by the neutrophil-to-lymphocyte count ratio. Isotope dilution liquid chromatography and tandem mass spectrometry were employed to measure serum testosterone concentration. Testosterone deficiency (TD) was defined as a serum testosterone level ≤ 300ng/dl. Weighted proportions and multivariable regression analyses were used to analyze the association between SII and TD.

**Results:**

Overall, the data of 7389 participants were analyzed, The SII ranged from 1.53 - 6297.60. Of the participants, 28.42% had a low serum testosterone level (≤ 300 ng/dl). In the fully adjusted multivariable logistic model, the second quartile (OR: 1.27, p = 0.0737), the third quartile (OR: 1.43, p = 0.0090), and the fourth quartile (OR:1.48, p = 0.0042) of SII significantly increased the TD incidence rate, with the lowest quartile of the SII as a reference. For subgroup analysis, statistically significant associations were observed in participants aged 20-40, obese, non-hypertensive, and non-diabetic. The interaction test revealed no significant effect on this connection.

**Conclusions:**

There was a positive relationship between a high SII and an increased prevalence of TD in a nationwide sample of adult men in the United States. Further prospective studies on a larger scale are warranted to confirm the causality between SII and TD.

## Introduction

Testosterone is a vital sexual hormone in men that is generated from the testes and the adrenal glands and is essential for reproduction and sexuality (1, 2). Testosterone deficiency (TD) is a highly common issue, and research has reported that it affects approximately 20% - 50% of American men (3, 4). Although TD can occur in men at any age, 7% of men develop TD after the age of 50, and this percentage increases as they get older (1). Symptoms caused by low testosterone levels can adversely lower a person’s quality of life both sexually and non-sexually. Low libido, erectile dysfunction, and difficulty in achieving orgasm are common symptoms (5). In addition, low energy, poor concentration, and depression were included. Furthermore, testosterone levels have been linked to muscle mass, bone strength, and iron metabolism. Thus, clinical attention should be paid to TD (1, 6).

Studies have examined the possible effect of the process of inflammation on testosterone and suggest that increased oxidative stress can lead to inflammation, which can negatively impact testosterone levels (7, 8). Bobjer et al. showed a correlation between low testosterone levels and pro-inflammatory mediators among relatively young men who had no metabolic diseases (9). A prevalence study conducted by Maggio et al. suggested a significantly negative correlation between testosterone and IL-6 in older men (10). Moreover, experimental studies have demonstrated that pro-inflammatory cytokines can control testosterone release by regulating the hypothalamic-pituitary-gonadal axis (11). These findings reiterated that increased levels of pro-inflammatory cytokines may cause an increase in TD.

The systemic immune-inflammation index (SII), A relatively novel inflammatory biomarker, is integrated with absolute blood counts of neutrophils, lymphocytes, and platelets (12). It has been identified as a better indicator of local and systemic inflammatory processes than other traditional factors in the human body. Several studies have reported that SII is associated with various malignant diseases, such as liver cancer (12), intestinal carcinoma (13), pancreatic carcinoma (14), carcinoma of the uterine cervix (15), and lung cancer (16). In addition, the SII has been proven to be a prognostic marker for major cardiovascular events. Yang et al. found that the higher the SII levels in CAD patients, the greater the risk of future cardiac death, nonfatal MI, and nonfatal stroke (17). Therefore, according to these studies, we believe that SII is an outstanding index of inflammation.

To date, research concerning the relationship between testosterone and inflammation has primarily concentrated on inflammatory factors, such as interleukin-1(IL-1), IL-6, and TNF-α (10, 18). However, the relationship between the SII and TD has not yet been assessed. Therefore, we evaluated the effect of SII on testosterone levels in participants of the National Health and Nutrition Examination Survey (NHANES) in the United States. We speculate that the incidence of TD will increase with an increase in the SII level.

## Materials and methods

### Data source and study population

**Figure 1:**
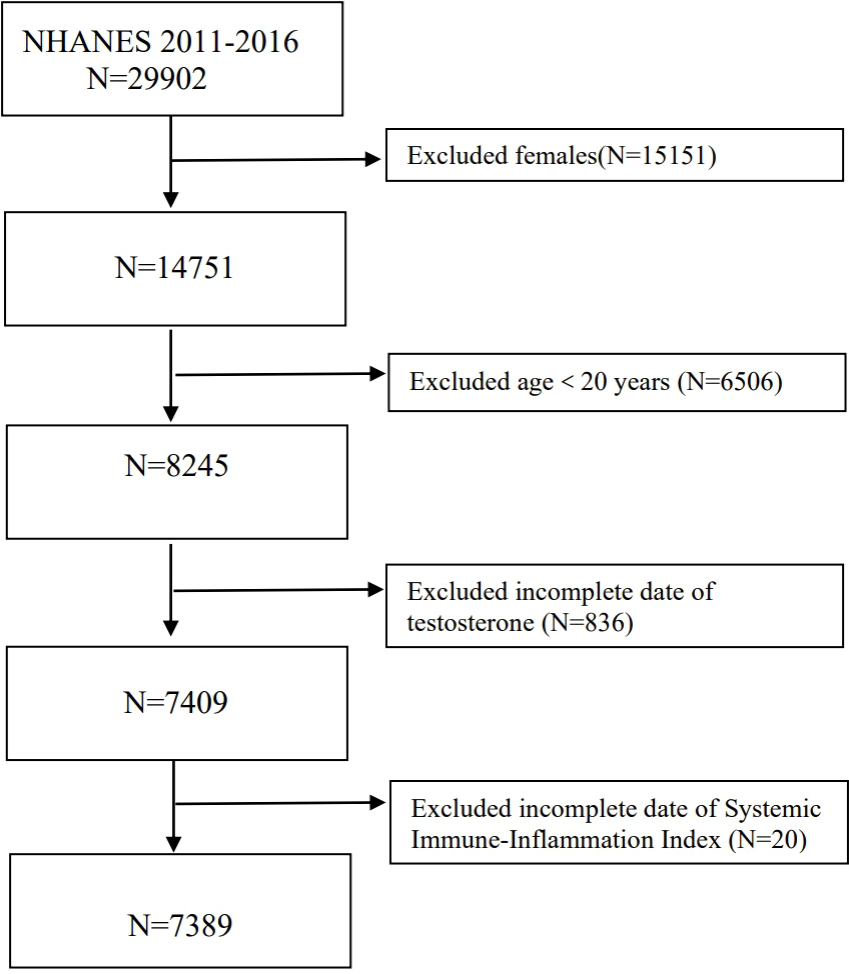
The selection process of NHANES 2011-2016.

Undertaken by the Centers for Disease Control and Prevention, the NHANES is used to evaluate the general health and nutritional status of the American population. All study participants provided written informed consent and underwent an ethical review by the National Center for Health Statistics Ethics Review Board. The Institutional Review Board of Northwestern University’s Feinberg School of Medicine determined that this study did not require a review. The data in this study were restricted to 2011-2012, 2013-2014, and 2015-2016 continuous data cycles. We excluded women, those aged <20 years, and those with insufficient data of SII and serum testosterone. Initially, the number of participants was 29902, women (n=15151), patients under 20 years old (n=6506), and those with missing data for serum testosterone (n=836) and SII (n=20) were excluded from the final analysis, Overall, 7389 qualified participants were included in our study (**Figure 1**). For more details on the survey design, methodology, and protocols, please visit the NHANES website (http://www.cdc.gov/nchs/nhanes.htm).

### Exposure and outcome definitions

SII, as an exposure variable, was obtained by calculating the total peripheral platelets count × neutrophil-to-lymphocyte ratio (N/L) (SII = P×N/L) (12). The serum total testosterone level in NHANES was determined by precise isotope dilution liquid chromatography and tandem mass spectrometry. The main consequence was the correlation between SII and serum total testosterone levels of ≤ 300ng/dl, following the guidelines of TD in the American Urological Association (19).

### Covariates

Based on a previous study (20), latent variables confounding the correlation between the SII and serum total testosterone were considered in the multivariable models. The covariates were age, body mass index (BMI), race, coronary artery sickness, heart failure, stroke, smoking status, alcohol consumption, educational qualifications, hypertension, diabetes, urine creatinine, triglycerides, and serum total cholesterol. The NHANES divides race/ethnicity into non-Hispanic white, non-Hispanic black, Mexican American, and other Hispanics. Qualifications were divided into grades 9 and below, grades 9 to 11, high school graduates, partial college or post-secondary qualifications, and college or higher qualifications. The participants were asked, “Have you ever smoked more than 100 cigarettes in your entire life?”, and were accordingly grouped into smokers and non-smokers based on their answers. BMI was categorized as <25, 25-29.9, or ≥30 kg/m^2^. Obesity was defined as a body mass index of ≥ 30. Participants who answered “yes” to the question “drinking at least 12 alcoholic beverages in a year” were considered to have a history of drinking. A history of high blood pressure can be determined by the answer: “Have you ever been told by a doctor that you had hypertension?” Defined diabetes as being “told by a doctor you have diabetes.” Urinary creatinine, triglycerides, and serum total cholesterol levels were derived from the standard biochemistry profiles.

### Statistical analysis

SII values were divided into quartiles for the logistic regression analysis to model the connection between quartile levels of SII and the odds of low testosterone levels. The lowest quartile was used as the reference for each case. Differences between categorical and continuous variables were evaluated using the Pearson’s chi-squared test and Student’s t-test for the SII groups. The mean ± standard deviation was used to express continuous variables, while proportions were used to present categorical variables. Using the generalized additive model (GAM), a nonlinear relation was identified. The association between the SII and TD was examined using multivariable logistic regression with three different models. Model 1 was not adjusted for covariates. In model 2, adjustments were made for age and race. Age, race, education level, BMI, coronary artery disease, heart failure, stroke, smoking status, alcohol consumption, hypertension, diabetes, urine creatinine, triglycerides, and serum cholesterol were adjusted in Model 3.We further applied four subgroups analyses on the connection of SII with TD with variables covering age (20-40/40-60/≥60 years), obesity (yes or no), hypertension (yes or no), and diabetes (yes or no) since these variables are known as prespecified possible effect modification. An interactive item was added to examine the correlations across groups. Statistical software packages R and Empower software (http://www.empowerstats.com, X&Y Solutions, Inc., Boston, MA) were used for all statistical analyses and NHANES sampling weights were considered. Odds ratios (ORs) and 95% confidence intervals (CI) were calculated for each target. Statistical significance was considered at a two-tailed p <0.05.

## Results

### Basic characteristics of the participants

A total of 7389 participants had complete data on SII and serum testosterone levels. **Table 1** lists the characteristics of the study population based on testosterone levels. The incidence of TD is 28.42. The SII of TD patients was significantly higher than that of non-TD patients (526.03 ± 308.27 vs 481.41 ± 318.79, p <0.001), Men presenting with normal total testosterone levels were relatively younger (p <0.001) and had a lower mean BMI (p <0.001), lower triglycerides (p <0.001), and lower urine creatinine (p=0.002) than men with lower total testosterone levels. Furthermore, participants with lower testosterone levels were more likely to develop diabetes, high blood pressure, heart failure, and coronary heart disease. The other socio-demographic differences were not significant (**Table 1**).

**Table 1:** Sociodemographic and clinical characteristics of the 7389 subjects related to the NHANES 2011–2016 cycle according to normal vs. low total testosterone level (ng/dl).

	Total testosterone		
	Normal (>300ng/dl)	Low (≤300ng/dl)	P-value
Sample size, n (%)	5289 (71.58%)	2100 (28.42%)	
Total testosterone, mean ± SD(ng/dl)	489.58 ± 167.71	222.25 ± 63.98	<0.001
Age, in years, mean ± SD	47.81 ± 17.65	52.68 ± 17.33	<0.001
Age, group class, n (%)			<0.001
20-40	2000 (37.81%)	556 (26.48%)	
40-60	1691 (31.97%)	725 (34.52%)	
60+	1598 (30.21%)	819 (39.00%)	
Race/ethnicity, n (%)			0.092
Mexican American	728 (13.76%)	295 (14.05%)	
Other Hispanic	522 (9.87%)	209 (9.95%)	
Non-Hispanic White	2020 (38.19%)	864 (41.14%)	
Non-Hispanic Black	1162 (21.97%)	416 (19.81%)	
Other Race	857 (16.20%)	316 (15.05%)	
BMI, mean ± SD (kg/m^2^)	27.42 ± 5.27	31.67 ± 7.00	<0.001
BMI group class, n (%)			<0.001
Normal(<25kg/m^2^)	1814 (34.30%)	275 (13.10%)	
Overweight (25-30 kg/m^2^)	2021 (38.21%)	706 (33.62%)	
Obese (30+ kg/m^2^)	1402 (26.51%)	1081 (51.48%)	
unknown	52 (0.98%)	38 (1.81%)	
Education level, n (%)			0.23
Less than 9th grade	515 (9.74%)	224 (10.67%)	
9th–11th grade	738 (13.95%)	284 (13.52%)	
High school graduate	1223 (23.12%)	484 (23.05%)	
Some college or AA degree	1456 (27.53%)	592 (28.19%)	
College graduate or above	1356 (25.64%)	513 (24.43%)	
unknown	1 (0.02%)	3 (0.14%)	
Creatinine, urine, mean ± SD (mg/dl)	138.95 ± 84.08	144.68 ± 82.89	0.002
SII, mean ± SD	481.41 ± 318.79	526.03 ± 308.27	<0.001
Total cholesterol, mean ± SD (mmol/L)	4.85 ± 1.06	4.84 ± 1.13	0.545
Triglycerides, mean ± SD (mmol/L)	1.39 ± 1.10	1.90 ± 1.74	<0.001
Smoking status, n (%)			0.361
Yes	2790 (52.75%)	1115 (53.10%)	
No	2495 (47.17%)	981 (46.71%)	
Unknown	4 (0.08%)	4 (0.19%)	
Alcohol consumption, n (%)			0.09
Yes	4094 (77.41%)	1587 (75.57%)	
No	793 (14.99%)	358 (17.05%)	
Unknown	402 (7.60%)	155 (7.38%)	
Hypertension, n (%)			<0.001
Yes	1702 (32.18%)	961 (45.76%)	
No	3579 (67.67%)	1138 (54.19%)	
Unknown	8 (0.15%)	1 (0.05%)	
Diabetes, n (%)			<0.001
Yes	585 (11.06%)	471 (22.43%)	
No	4701 (88.88%)	1628 (77.52%)	
Unknown	3 (0.06%)	1 (0.05%)	
Heart failure, n (%)			<0.001
Yes	138 (2.61%)	126 (6.00%)	
No	5144 (97.26%)	1968 (93.71%)	
Unknown	7 (0.13%)	6 (0.29%)	
Coronary artery disease, n (%)			<0.001
Yes	213 (4.03%)	163 (7.76%)	
No	5050 (95.48%)	1926 (91.71%)	
Unknown	26 (0.49%)	11 (0.52%)	
Stroke, n (%)			0.423
Yes	182 (3.44%)	84 (4.00%)	
No	5105 (96.52%)	2015 (95.95%)	
Unknown	2 (0.04%)	1 (0.05%)	

BMI, body mass index; SII, systemic immune-inflammation index.

### Association between SII and increased serum testosterone

**Figure 2:**
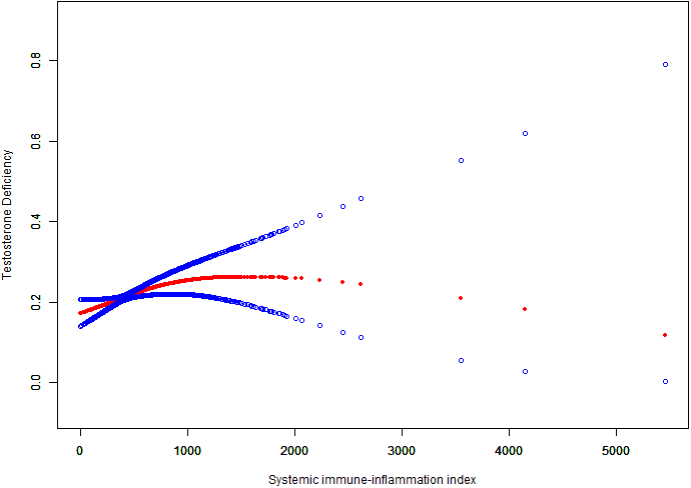
The Nonlinear relationship between SII and TD incidence rate. A smooth curve fitting between variables is represented by the solid red line. The 95% confidence interval of the fit is indicated by the blue line.The values were adjusted for age, race, BMI, coronary artery disease, heart failure, stroke, smoking status, alcohol consumption, education level, hypertension, diabetes, triglycerides, urine creatinine, and serum total cholesterol.

Smooth curve fitting showed that after adjusting for age, BMI, race, coronary artery disease, heart failure, stroke, smoking status, alcohol consumption, education level, hypertension, diabetes, urine creatinine, triglycerides, and serum total cholesterol, SII not linearly associated with TD prevalence (**Figure 2**). Then we performed a two-piecewise linear regression model to calculate the threshold effect of the SII on TD according to the smoothing plot. we found the inflection points were 742.5. On the left of the inflection point, the P value was 0.0019. However, we observed no relationship between SII and TD on the right of the inflection point (P>0.05) (**Supplementary Table 1**). We then conduct a logistic regression analysis by transforming the SII from a continuous variable into a classified variable (quartile 1:1.53-302.4, quartile 2:302.50-424.53, quartile 3:424.63-601.33, quartile 4:601.59-6297.60). As is shown in **Table 2**, a strong positive correlation existed between the SII quartiles and TD, and the OR values of TD increased with an increase in SII in the non-adjusted logistic regression model (OR=1.60; 95% CI, 1.38–1.84, p<0.0001). This meaningful relevance also exists in the adjusted I-model. In the fully adjusted model (Adjusted II), the OR values of TD in the highest SII quartile were significantly higher than those in the lowest quartile (OR=1.48; 95% CI, 1.13–1.93, p=0.0042) (**Table 2**).

**Table 2:** Logistic regression analysis was used to assess the correlation between the prevalence of testosterone deficiency and SII.

	OR (95%CI), p-value		
	Non-adjusted^b^	Adjust I^c^	Adjust II^d^
SII^a^ group			
Quartile 1(Q1)	Reference	Reference	Reference
Quartile 2(Q2)	1.15 (0.99, 1.33) <0.0744	1.13 (0.97, 1.32) 0.1089	1.27 (0.98, 1.64) 0.0737
Quartile 3(Q3)	1.40 (1.21, 1.62) <0.0001	1.37 (1.18, 1.59) <0.0001	1.43 (1.09, 1.87) 0.0090
Quartile 4(Q4)	1.60 (1.38, 1.84) <0.0001	1.46 (1.26, 1.70) <0.0001	1.48 (1.13, 1.93) 0.0042
SII group trend	<0.0001	<0.0001	0.006

CI, confidence interval; OR, odds ratio.

^a^Presented in quartiles.

^b^Non-adjusted model adjusts for None.

^c^Adjust I model adjust for age, race.

^d^Adjust II model adjusts for age, race, BMI, coronary artery disease, heart failure, stroke, smoking status, alcohol consumption, education level, hypertension, diabetes, triglycerides, urine creatinine, and serum total cholesterol.

### Subgroup analysis

**Figure 3:**
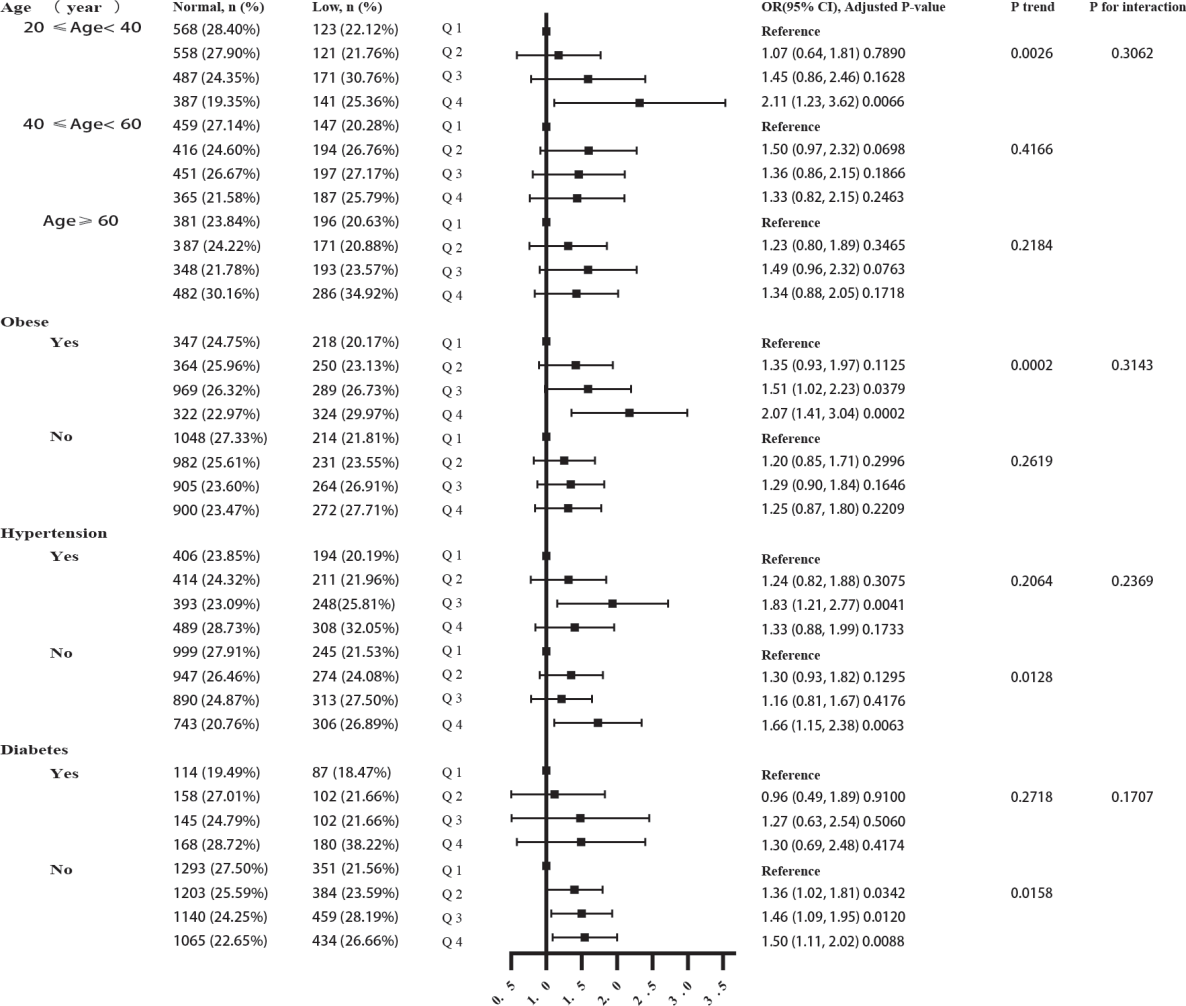
The forest plot of correlation subgroup analysis between SII and TD.

After modulating all possible confounding factors, we found that participants in quartile 4 developing TD was 2.07 times that of the quartile 1 among obese group (OR 2.07; 95% CI 1.41-3.04; p =0.0002). No statistically significant association was observed among men with normal weight. Meanwhile, only the 20-40 age group had a significant correlation, and the OR value of the highest SII quartile reporting TD was 2.11 times that of the lowest SII quartile (OR=2.11; 95%CI, 1.23-3.62, p= 0.0066). However, for participants aged 40-60, the OR value of quartile 2 reporting TD was 1.50 times that of the lowest SII quartile (OR=1.50; 95%CI, 0.97-2.32, p= 0.0698); for participants with aged ≥ 60 years, the OR value of quartile 3 reporting TD was 1.49 times that of the lowest SII quartile (OR=1.49; 95%CI, 0.96-2.32, p= 0.0763). Both two associations were close to meet the statistical significance. Moreover, participants in quartile 4 had a 66% higher risk of TD (OR 1.66; 95% CI 1.15-2.38; p =0.0063) than men without hypertension in quartile 1. Furthermore, participants in quartile 4 had a 50% higher risk of TD (OR 1.50; 95% CI 1.11-2.02; p =0.0088) than men without diabetes in quartile 1. The interaction test revealed that no significant correlation existed between SII and TD among all levels, suggesting that there was no significant dependence on age, obesity, high blood pressure, or diabetes (all p for interaction >0.05) (**Figure 3**).

## Discussion

TD, also known as hypogonadism, due to testicular dysfunction or hypothalamic-pituitary dysfunction and it can either develop congenitally or acquired (5). TD is often overlooked because of its insidious and nonspecific clinical presentation (21). Evidence suggests that TD is associated with a variety of diseases, including obesity, cardiovascular disease, and depression. The association between inflammation and testosterone has been widely investigated since chronic inflammation is one of the pathogeneses of numerous diseases (8, 22). According to recent research, testosterone plays a vital role in regulating inflammation (23). For example, as a common urological disease, the progression of benign prostatic hyperplasia involves chronic inflammation and immune disorders (24). The etiopathogenesis of benign prostate hyperplasia is believed to be the infiltration of activated CD4+ T lymphocytes and the release of inflammatory cytokines (25). In 2009, Filippi S et al. established an animal model of hypogonadism induced by a high-fat diet (26). They found that the histological changes in the prostate were inflammation and stromal disorder, and such changes could be completely reversed by testosterone supplementation (26). Subsequently, they conducted a clinical retrospective study, by observing the prostatectomy specimens from patients with benign prostate hyperplasia in the histological characteristics of inflammatory infiltration (27). They found that the inflammatory infiltration and testosterone levels were significantly negatively correlated. Moreover, hypogonadism increased the risk of prostate gland inflammation by five times, and the *in vitro* experiment revealed that dihydrotestosterone had extensive anti-inflammatory effects on testosterone cells (27). An animal experiment by Monika F et al. showed evidence for an immunomodulatory and protective effect of testosterone by regulating the differentiation of T lymphocytes (28). In contrast, excessive inflammation can in turn modulate the hypothalamic-pituitary-gonadal axis and restrain testosterone release (7, 11).

To the best for our knowledge, the relationship between SII and TD has not been assessed. In this cross-sectional study with 7389 enrolled participants, the relationship between SII and serum testosterone levels was investigated in men and to determine whether this relationship varies with age, obesity, hypertension, and diabetes. The mean SII of participants with TD was significantly higher than that of men without TD. In addition, we discovered that men with a higher SII had an increased risk of TD in an unadjusted logistic regression model. This connection is consistent even after adjusting for all possible confounders; compared to quartile 1, the risk of TD was relatively higher in quartile 4. Notably, when we restricted the participants to obese men, the risk of TD was significant. Obese patients had a higher risk of TD than non-obese patients, suggesting that a higher SII accelerates testosterone decline in obese patients. These results further support an endocrine-disrupting role of inflammation. Epidemiological surveys have shown that total testosterone in the blood is inversely related to obesity (29), and the prevalence of TD is up to 79% in obese individuals (30). Chronic inflammation is considered the main reason for low testosterone levels in the obese population because in a state of inflammation, inflammatory cytokines are mainly derived from adipose tissue, which can reduce testosterone production by converting testosterone to estradiol (31). Furthermore, adipose tissue also generates leptin, which negatively affects the hypothalamic-pituitary-gonadal axis by inhibiting gonadotropins on testicular interstitial cells, leading to a decrease in androgen production (32). Interestingly, after adjusting for confounding factors in the subgroup analysis, we noted that there was no correlation between TD and SII in people over 40 years of age; however, a significant correlation was observed in people aged 20-40 years. Aging in men is accompanied by a decrease in serum testosterone secretion. It has been reported that testosterone levels in men drop by approximately 1–1.6% per year after the age of 40 years (33). However, men aged 20-40 year have a normal gonadal function and rarely have metabolic diseases. Thus, we believe that SII is directly related to the incidence of TD in men aged 20-40 years. Bobjer et al. analyzed the changes of a series of inflammatory markers in 60 young men, with the purpose of investigating the relationship between low testosterone levels and inflammation in youth (9). These results indicated that low testosterone levels were significantly linked to the pro-inflammatory chemokines in young men, which corroborated our present findings. Unexpectedly, an association between SII and TD among non-hypertensive and non-diabetic men was observed, rather than among hypertensive and diabetic men. We attributed this result to the influence of antihypertensive and hypoglycemic drugs on testosterone levels. Numerous studies have shown that the use of hypotensive (34–36) and hypoglycemic drugs (37) can affect testosterone levels. For example, the antihypertensive drug β-blockers atenolol, can inhibit the sympathetic nervous system, thereby inhibiting the release of testosterone (38, 39). In contrast, angiotensin-converting enzyme inhibitors (40)and angiotensin II receptor blockers (41), such as captopril, have been associated with improved sexual function. Studies have shown that the use of metformin, a first-line glucose-lowering drug for the treatment of type 2 diabetes, also reduces serum testosterone levels in patients with diabetes (37). Patients with type 2 diabetes had significantly lower testosterone levels after three month of metformin treatment (42). So we think that drug use in patients with hypertension and diabetes may confuse the relationship between the SII and TD.

The results of our study are consistent with the previous views because several research has been done on the relationship between testosterone and inflammatory responses and has proven that elevated levels of inflammation can cause a decline in testosterone levels (43, 44). A review performed by Mohamad explored the effect and mechanism of testosterone on the inflammatory response by summarizing all the evidence from human observation and animal research that reported the relationship between testosterone and inflammatory markers (8). This review suggests that TD can promote an elevation of inflammatory factor levels. In addition, anti-inflammatory activity may be a function of testosterone in the body. Testosterone therapy can counteract pro-inflammatory insults, significantly improving MetS-induced hypertension, visceral adipose tissue accumulation, and glucose homeostasis derangements, and also has anti-inflammatory effects at the hypothalamic level (45). However, the outcome remains dependent on the anti-inflammatory effects of testosterone. Ebrahimi et al. conducted a parallel-group, randomized trial from 2016 to 2017, wherein 33 and 34 patients received recombinant human IL-1 receptors and placebo, respectively. The results showed that the use of IL-1 receptor antagonists increased testosterone levels in obese individuals (46). An NHANES study conducted by Zhang et al. investigated the association between the dietary inflammatory index and male hormones and reported that men who adhere to an inflammatory diet appear to be at greater risk for TD (20). Compared with traditional inflammatory factors, the SII better reflects the inflammatory state and has shown better prognostic value in several studies. The SII may be an easily accessible and cost-effective strategy to identify low testosterone levels in men, and more attention should be paid to young men and obese people. However, our study has several limitations. As a prevalence study database, we could not confirm the causal relationship between the SII and TD, which is the main restriction. Secondly, some residual confounders, such as prostate cancer, physical activity, and hormone medication, may still affect the association between SII and testosterone. According to previous researches (47, 48), obesity is associated with low total testosterone it is common that free testosterone is normal due to lower SHBG, the relationship between SHBG and free testosterone and SII should be evaluated in future study. Finally, according to the American Urological Association guidelines, serum testosterone should be measured twice and conducted in an early morning fashion (19).

In summary, our research showed that increased SII levels are associated with an increased prevalence of TD. Broader and deeper studies are needed to verify our results.

## Data availability statement

The datasets presented in this study can be found in online repositories. The names of the repository/repositories and accession number(s) can be found below: https://www.cdc.gov/nchs/nhanes/.

## Ethics statement

The studies involving human participants were reviewed and approved by The Wake Forest School of Medicine Institutional Review Board. The patients/participants provided their written informed consent to participate in this study.

## Author contributions

YL: data analysis, writing an original draft. ML: investigation, resources. YC: software, investigation. ZZ: methodology, resources. JC: writing review, formal analysis. FZ: methodology, software, formal analysis. MG: methodology, software. LY: conceptualization. FH: conceptualization and writing reviewing and editing. HC: conceptualization, project administration, supervision. It has been approved for publication by all the authors listed.

## Funding

The National Natural Science Foundation of China provided funding for this study (82170781).

## Acknowledgments

Special thanks to Dr. Fang Huang for reviewing the manuscript.

## Conflict of interest

The authors declare that the research was conducted in the absence of any commercial or financial relationships that could be construed as a potential conflict of interest.

## Publisher’s note

All claims expressed in this article are solely those of the authors and do not necessarily represent those of their affiliated organizations, or those of the publisher, the editors and the reviewers. Any product that may be evaluated in this article, or claim that may be made by its manufacturer, is not guaranteed or endorsed by the publisher.

## Abbreviations

BMI, Body mass index; CI, Confidence intervals; IL, Interleukin; NHANES, National Health and Nutrition Examination Survey; ORs, Odds ratios; SII, Systemic immune-inflammation index; TD, Testosterone deficiency.

## References

[1] Halpern JA Brannigan RE. Testosterone deficiency. JAMA (2019) 322(11):1116. doi: 10.1001/jama.2019.9290 31529009

[2] Allen NE Key TJ. The effects of diet on circulating sex hormone levels in men. Nutr Res Rev (2000) 13(2):159–84. doi: 10.1079/095442200108729052 19087438

[3] Araujo AB O'Donnell AB Brambilla DJ Simpson WB Longcope C Matsumoto AM. Prevalence and incidence of androgen deficiency in middle-aged and older men: estimates from the Massachusetts Male aging study. J Clin Endocrinol Metab (2004) 89(12):5920–6. doi: 10.1210/jc.2003-031719 15579737

[4] Mulligan T Frick MF Zuraw QC Stemhagen A McWhirter C. Prevalence of hypogonadism in males aged at least 45 years: the HIM study. Int J Clin Pract (2006) 60(7):762–9. doi: 10.1111/j.1742-1241.2006.00992.x PMC1569444 16846397

[5] Aversa A Morgentaler A. The practical management of testosterone deficiency in men. Nat Rev Urol (2015) 12(11):641–50. doi: 10.1038/nrurol.2015.238 26458755

[6] Beattie MC Adekola L Papadopoulos V Chen H Zirkin BR. Leydig cell aging and hypogonadism. Exp Gerontol (2015) 68:87–91. doi: 10.1016/j.exger.2015.02.014 25700847 PMC5662440

[7] Bini EI D'Attilio L Marquina-Castillo B Mata-Espinosa D Diaz A Marquez-Velasco R. The implication of pro-inflammatory cytokines in the impaired production of gonadal androgens by patients with pulmonary tuberculosis. Tuberculosis (Edinb) (2015) 95(6):701–6. doi: 10.1016/j.tube.2015.06.002 26602224

[8] Mohamad NV Wong SK Wan Hasan WN Jolly JJ Nur-Farhana MF Ima-Nirwana S. The relationship between circulating testosterone and inflammatory cytokines in men. Aging Male (2019) 22(2):129–40. doi: 10.1080/13685538.2018.1482487 29925283

[9] Bobjer J Katrinaki M Tsatsanis C Lundberg Giwercman Y Giwercman A. Negative association between testosterone concentration and inflammatory markers in young men: a nested cross-sectional study. PloS One (2013) 8(4):e61466. doi: 10.1371/journal.pone.0061466 23637840 PMC3630214

[10] Maggio M Basaria S Ble A Lauretani F Bandinelli S Ceda GP. Correlation between testosterone and the inflammatory marker soluble interleukin-6 receptor in older men. J Clin Endocrinol Metab (2006) 91(1):345–7. doi: 10.1210/jc.2005-1097 16263825

[11] Darbandi M Darbandi S Agarwal A Sengupta P Durairajanayagam D Henkel R. Reactive oxygen species and male reproductive hormones. Reprod Biol Endocrinol (2018) 16(1):87. doi: 10.1186/s12958-018-0406-2 30205828 PMC6134507

[12] Hu B Yang XR Xu Y Sun YF Sun C Guo W. Systemic immune-inflammation index predicts prognosis of patients after curative resection for hepatocellular carcinoma. Clin Cancer Res (2014) 20(23):6212–22. doi: 10.1158/1078-0432.CCR-14-0442 25271081

[13] Chen JH Zhai ET Yuan YJ Wu KM Xu JB Peng JJ. Systemic immune-inflammation index for predicting prognosis of colorectal cancer. World J Gastroenterol (2017) 23(34):6261–72. doi: 10.3748/wjg.v23.i34.6261 PMC5603492 28974892

[14] Murthy P Zenati MS Al Abbas AI Rieser CJ Bahary N Lotze MT. Prognostic value of the systemic immune-inflammation index (SII) after neoadjuvant therapy for patients with resected pancreatic cancer. Ann Surg Oncol (2020) 27(3):898–906. doi: 10.1245/s10434-019-08094-0 31792715 PMC7879583

[15] Huang H Liu Q Zhu L Zhang Y Lu X Wu Y. Prognostic value of preoperative systemic immune-inflammation index in patients with cervical cancer. Sci Rep (2019) 9(1):3284. doi: 10.1038/s41598-019-39150-0 30824727 PMC6397230

[16] Alifano M. Systemic immune-inflammation index and prognosis of advanced non-small cell lung cancer. Ann Transl Med (2020) 8(11):667. doi: 10.21037/atm.2020.03.174 32617287 PMC7327370

[17] Yang YL Wu CH Hsu PF Chen SC Huang SS Chan WL. Systemic immune-inflammation index (SII) predicted clinical outcome in patients with coronary artery disease. Eur J Clin Invest (2020) 50(5):e13230. doi: 10.1111/eci.13230 32291748

[18] Wang M Tsai BM Kher A Baker LB Wairiuko GM Meldrum DR. Role of endogenous testosterone in myocardial proinflammatory and proapoptotic signaling after acute ischemia-reperfusion. Am J Physiol Heart Circ Physiol (2005) 288(1):H221–6. doi: 10.1152/ajpheart.00784.2004 15374831

[19] Mulhall JP Trost LW Brannigan RE Kurtz EG Redmon JB Chiles KA. Evaluation and management of testosterone deficiency: AUA guideline. J Urol (2018) 200(2):423–32. doi: 10.1016/j.juro.2018.03.115 29601923

[20] Zhang C Bian H Chen Z Tian B Wang H Tu X. The association between dietary inflammatory index and sex hormones among men in the united states. J Urol (2021) 206(1):97–103. doi: 10.1097/JU.0000000000001703 33881929

[21] Corona G Rastrelli G Vignozzi L Mannucci E Maggi M. How to recognize late-onset hypogonadism in men with sexual dysfunction. Asian J Androl (2012) 14(2):251–9. doi: 10.1038/aja.2011.138 PMC3735094 22286862

[22] Grandys M Majerczak J Zapart-Bukowska J Duda K Kulpa JK Zoladz JA. Lowered serum testosterone concentration is associated with enhanced inflammation and worsened lipid profile in men. Front Endocrinol (Lausanne) (2021) 12:735638. doi: 10.3389/fendo.2021.735638 34566895 PMC8459752

[23] Rettew JA Huet-Hudson YM Marriott I. Testosterone reduces macrophage expression in the mouse of toll-like receptor 4, a trigger for inflammation and innate immunity. Biol Reprod (2008) 78(3):432–7. doi: 10.1095/biolreprod.107.063545 18003947

[24] Fibbi B Penna G Morelli A Adorini L Maggi M. Chronic inflammation in the pathogenesis of benign prostatic hyperplasia. Int J Androl (2010) 33(3):475–88. doi: 10.1111/j.1365-2605.2009.00972.x 19508330

[25] Kramer G Mitteregger D Marberger M. Is benign prostatic hyperplasia (BPH) an immune inflammatory disease? Eur Urol (2007) 51(5):1202–16. doi: 10.1016/j.eururo.2006.12.011 17182170

[26] Filippi S Vignozzi L Morelli A Chavalmane AK Sarchielli E Fibbi B. Testosterone partially ameliorates metabolic profile and erectile responsiveness to PDE5 inhibitors in an animal model of male metabolic syndrome. J Sex Med (2009) 6(12):3274–88. doi: 10.1111/j.1743-6109.2009.01467.x 19732305

[27] Vignozzi L Cellai I Santi R Lombardelli L Morelli A Comeglio P. Antiinflammatory effect of androgen receptor activation in human benign prostatic hyperplasia cells. J Endocrinol (2012) 214(1):31–43. doi: 10.1530/JOE-12-0142 22562653

[28] Fijak M Schneider E Klug J Bhushan S Hackstein H Schuler G. Testosterone replacement effectively inhibits the development of experimental autoimmune orchitis in rats: evidence for a direct role of testosterone on regulatory T cell expansion. J Immunol (2011) 186(9):5162–72. doi: 10.4049/jimmunol.1001958 21441459

[29] Kelly DM Jones TH. Testosterone and obesity. Obes Rev (2015) 16(7):581–606. doi: 10.1111/obr.12282 25982085

[30] Pellitero S Olaizola I Alastrue A Martinez E Granada ML Balibrea JM. Hypogonadotropic hypogonadism in morbidly obese males is reversed after bariatric surgery. Obes Surg (2012) 22(12):1835–42. doi: 10.1007/s11695-012-0734-9 22923309

[31] Calder PC Ahluwalia N Brouns F Buetler T Clement K Cunningham K. Dietary factors and low-grade inflammation in relation to overweight and obesity. Br J Nutr (2011) 106 Suppl 3:S5–78. doi: 10.1017/S0007114511005460 22133051

[32] Fui MN Dupuis P Grossmann M. Lowered testosterone in male obesity: mechanisms, morbidity and management. Asian J Androl (2014) 16(2):223–31. doi: 10.4103/1008-682X.122365 PMC3955331 24407187

[33] Kaufman JM Vermeulen A. The decline of androgen levels in elderly men and its clinical and therapeutic implications. Endocr Rev (2005) 26(6):833–76. doi: 10.1210/er.2004-0013 15901667

[34] Manolis A Doumas M. Antihypertensive treatment and sexual dysfunction. Curr Hypertens Rep (2012) 14(4):285–92. doi: 10.1007/s11906-012-0276-5 22581395

[35] Manolis A Doumas M Ferri C Mancia G. Erectile dysfunction and adherence to antihypertensive therapy: Focus on beta-blockers. Eur J Intern Med (2020) 81:1–6. doi: 10.1016/j.ejim.2020.07.009 32693940

[36] Al Khaja KA Sequeira RP Alkhaja AK Damanhori AH. Antihypertensive drugs and Male sexual dysfunction: A review of adult hypertension guideline recommendations. J Cardiovasc Pharmacol Ther (2016) 21(3):233–44. doi: 10.1177/1074248415598321 26450998

[37] Hu Y Ding B Shen Y Yan RN Li FF Sun R. Rapid changes in serum testosterone in men with newly diagnosed type 2 diabetes with intensive insulin and metformin. Diabetes Care (2021) 44(4):1059–61. doi: 10.2337/dc20-1558 PMC7985426 33536253

[38] Barksdale JD Gardner SF. The impact of first-line antihypertensive drugs on erectile dysfunction. Pharmacotherapy (1999) 19(5):573–81. doi: 10.1592/phco.19.8.573.31526 10331820

[39] Fogari R Preti P Derosa G Marasi G Zoppi A Rinaldi A. Effect of antihypertensive treatment with valsartan or atenolol on sexual activity and plasma testosterone in hypertensive men. Eur J Clin Pharmacol (2002) 58(3):177–80. doi: 10.1007/s00228-002-0456-3 12107602

[40] Baumhakel M Schlimmer N Bohm M Investigators D-I. Effect of irbesartan on erectile function in patients with hypertension and metabolic syndrome. Int J Impot Res (2008) 20(5):493–500. doi: 10.1038/ijir.2008.28 18596705

[41] Dusing R. Effect of the angiotensin II antagonist valsartan on sexual function in hypertensive men. Blood Press Suppl (2003) 2:29–34. doi: 10.1080/08038020310021967 14761074

[42] Cai T Hu Y Ding B Yan R Liu B Cai L. Effect of metformin on testosterone levels in Male patients with type 2 diabetes mellitus treated with insulin. Front Endocrinol (Lausanne) (2021) 12:813067. doi: 10.3389/fendo.2021.813067 35002984 PMC8740051

[43] Barbosa LP da Silva Aguiar S Santos PA Dos Santos Rosa T Maciel LA de Deus LA. Relationship between inflammatory biomarkers and testosterone levels in male master athletes and non-athletes. Exp Gerontol (2021) 151:111407. doi: 10.1016/j.exger.2021.111407 34022273

[44] Vodo S Bechi N Petroni A Muscoli C Aloisi AM. Testosterone-induced effects on lipids and inflammation. Mediators Inflamm (2013) 2013:183041. doi: 10.1155/2013/183041 23606790 PMC3628213

[45] Sarchielli E Comeglio P Filippi S Cellai I Guarnieri G Marzoppi A. Neuroprotective effects of testosterone in the hypothalamus of an animal model of metabolic syndrome. Int J Mol Sci (2021) 22(4):1589. doi: 10.3390/ijms22041589 33557413 PMC7914611

[46] Ebrahimi F Urwyler SA Straumann S Doerpfeld S Bernasconi L Neyer P. IL-1 antagonism in men with metabolic syndrome and low testosterone: A randomized clinical trial. J Clin Endocrinol Metab (2018) 103(9):3466–76. doi: 10.1210/jc.2018-00739 29939279

[47] Rastrelli G O'Neill TW Ahern T Bartfai G Casanueva FF Forti G. Symptomatic androgen deficiency develops only when both total and free testosterone decline in obese men who may have incident biochemical secondary hypogonadism: Prospective results from the EMAS. Clin Endocrinol (Oxf) (2018) 89(4):459–69. doi: 10.1111/cen.13756 29855071

[48] Antonio L Wu FC O'Neill TW Pye SR Ahern TB Laurent MR. Low free testosterone is associated with hypogonadal signs and symptoms in men with normal total testosterone. J Clin Endocrinol Metab (2016) 101(7):2647–57. doi: 10.1210/jc.2015-4106 26909800

