# Supplementary material for: Increased risk of testosterone deficiency is associated with the systemic immune-inflammation index: a population-based cohort study
Source: Front Endocrinol (Lausanne). 2022 Aug 16;13:974773. doi: 10.3389/fendo.2022.974773 (PMC9424499; doi:10.3389/fendo.2022.974773)
Supplement: Supplementary file 1 [file Table_1.docx]

| Supplementary Table 1 Threshold effect analysis of SII on the prevalence of TD using piece-wise linear regression. | | | |
| --- | --- | --- | --- |
| Inflection points | Adjusted OR (95%CI) p-value | | |
| <742.5 | 1.0008 (1.0003,1.0014) |  | 0.0019 |
| ≥742.5 | 0,9998 (0.9992,1.0003) |  | 0.4010 |
